# Caloric restriction increases ratio of estrogen to androgen receptors expression in murine ovaries - potential therapeutic implications

**DOI:** 10.1186/s13048-015-0185-8

**Published:** 2015-08-13

**Authors:** Sylwia Słuczanowska-Głąbowska, Maria Laszczyńska, Katarzyna Piotrowska, Marta Grabowska, Katarzyna Grymuła, Mariusz Z. Ratajczak

**Affiliations:** Department of Physiology Pomeranian Medical University, Powstańców Wielkopolskich 72, 70-111 Szczecin, Poland; Department of Histology and Developmental Biology, Pomeranian Medical University, Żołnierska 48, 71-210 Szczecin, Poland; Stem Cell Biology Program, James Graham Brown Cancer Center, University of Louisville, Louisville, Ky USA

**Keywords:** Ovary, Mice, Androgen receptor, Estrogen receptors, Caloric restriction

## Abstract

Both estrogens and androgens are involved in the development and normal functioning of the ovaries. It is also known that ovarian function is regulated by diet. The goal of this study was to estimate the expression of sex hormone receptors in ovaries of mice that were on a 9-month caloric restriction (alternate-day feeding) as compared to normal control animals fed *ad libitum*. We found that prolonged caloric restriction in mouse ovaries led to increased expression of estrogen receptors (ERs) but did not affect expression of the androgen receptor (AR). This increase in ER:AR ration as result of caloric restriction may lead to higher sensitivity to estrogens and upon return to normal diet may increase ovulation. Thus our observation shed more light on a role of beneficial effect of calorie restriction on female reproduction.

## Introduction

Estrogens and androgens are involved in the development and normal functioning of the ovaries. Estrogen, which is mainly synthesized in the ovary, is also involved in the development and functioning of other components of the female reproductive system. It is primarily expressed by preovulatory follicles under the influence of FSH [1]. In healthy premenopausal women, the main estrogen, 17β-estradiol, is produced in the ovaries. In postmenopausal women and in men, 17β-estradiol is produced in extra gonadal sites, mainly by aromatization of circulating testosterone in adipose tissue [2, 3]. The main function of estrogen in females is to control the estrous cycle and regulate development and maturation of the reproductive tract. Ovarian-produced estrogens are essential for cell proliferation, follicular development, and growth as well as follicular atresia. Estrogens also augment the action of follicle-stimulating hormone (FSH), increase responsiveness of ovaries to gonadotropins, increase aromatase activity, and estrogen synthesis [1–5].

The biological effects of estrogen are mediated by two nuclear estrogen receptors (ERs): ERα and ERβ. In the ovary, ERβ expression is predominant. In immature and adult animals, ERβ is found in the nuclei of granulosa cells of primary, secondary, and mature follicles, and a weak immunoreactive response is also detected in atretic follicle granulosa cells. Immunoreactivity for ERβ was also found in theca cells, luteal cells, interstitial cells, ovarian surface epithelium, and oocytes. By contrast, ERα is present in theca cells, interstitial cells, and in ovarian surface epithelium; low expression is noted in granulosa cells [1, 4, 6, 7]. It is interesting that the number of ERα receptors is significantly reduced in animals in the nonbreeding season [1]. It has also been observed that, while ERα knockout female mice are infertile and do not ovulate, ERβ knockout female mice display impaired fertility, with reduced litter numbers and smaller litter size compared with wild type mice [5].

The androgens play an important regulatory role in the ovaries during follicular development and are responsible for follicle initiation and early growth [1, 8]. The main androgen, androstendione, is synthesized from progestagens in theca cells under the influence of luteinizing hormone (LH) [9–11]. The androgens appear to be capable of improving the early stages of folliculogenesis in ovaries. In mammals, including in human, androgens are converted into estrogen, which regulates follicular development via binding to ERs during the reproductive life span. In aged mammals, only a small portion of androgens is converted into estrogens, and the androgens predominantly bind to the androgen receptor (AR) and may affect follicular development [8–13].

The ovarian AR is localized mainly in granulosa cells but is also found in oocytes, theca, and interstitial cells [7, 14–16]. The follicles in the early stages of development express a larger number of AR proteins than those in more advanced stages. It is significant that expression of AR mRNA increases as the follicles make the transition from primordial to preantral follicles, and the expression of AR is highest in preantral follicles, gradually decreasing as the follicles mature. The AR and its mRNA are developmentally regulated and are down-regulated during FSH-stimulated preovulatory follicular development [7, 8, 14–17]. Granulosa cells in preantral and antral follicles constitute a crucial site for AR-mediated action involved in maintaining follicle and embryo survival and optimal female fertility. The expression of the AR is higher in the ovaries of old female animals and postmenopousal women than in reproductive age women and animals in nonbreeding season [1, 7, 8, 12–19]. In seasonally breeding animals, androgens play a crucial role in the transition from the breeding to the nonbreeding season and regulate follicular atresia. Animals in the breeding season exhibit all stages of follicular development, but in the nonbreeding season only preantral follicles are present [1].

It is well known that nutrition and energy metabolism influence reproductive function [20]. In human and other mammals, malnutrition delays the onset of puberty and leads to ovulation problems and embryonic mortality. Nutrients and metabolic hormones, including insulin and insulin-like growth factor 1 (IGF-1), are needed to maintain normal function of the hypothalmus–pituitary axis and also to maintain the reproductive cells in the gonads [20–23].

Caloric restriction (CR) is one of the factors that extends life span, and it minimizes the age-related dysfunction of many organs, including those of the reproductive system [24, 25]. It is known that decreased food intake leads to slower growth but also increased longevity [21, 24, 26]. Selesniemi et al. [22] showed that CR delays sexual maturation, extends female fertile lifespan, and leads to maintenance of the ovarian follicle reserve. Gutierrez et al. [27] showed that an increase in dietary intake leads to increased recruitment of small follicles during the first follicular wave of the estrous cycle but not to follicle selection and dominance. Armstrong et al. [28] observed a dietary-induced increase in aromatase activity in small follicles. Nelson et al. [29] reported that CR delays the age-related loss of cycling capacity, the age-related increase in cycle length, and the age-related loss of primordial follicles [29].

Our recent morphological study [30] showed that the ovaries of 2.5-year-old wild type (WT) mice on CR appeared to be younger than 2.5-year-old wild type mice fed *ad libitum*. While ovaries of WT mice on CR still contained some follicles at different stages of development, ovaries in 2.5-year-old WT mice fed *ad libitum* were depleted of follicles. Similarly, we observed that the ovaries of 10-month-old female mice on CR had higher numbers of primordial, primary, and preantral follicles than the ovaries of mice fed *ad libitum* [25]. We also examined male mice on CR and we observed a ~25 % reduction in body weight in male mice on CR [25].

To better explain these observations, we examined the expression of ER and AR receptors in the ovaries of mice on CR compared with mice fed *ad libitum*.

## Materials and methods

### Animals

This study was undertaken in the Department of Physiology Pomeranian Medical University in Szczecin in accordance with the guidelines of the Local Ethical Committee (Use of Laboratory Animals) in Szczecin, Poland.

The experiment was performed on one-month-old C57B1/6 female mice. Twelve mice were divided into two groups: control (n = 6) and experimental (n = 6). The animals of the control group were given free access to a nutritionally balanced diet in doses of 7 g/day/per mouse (Murigan, Motycz, Poland) *ad libitum*. The animals of the experimental group on caloric restriction (CR) were fed *ad libitum* every other day at the same dose (7 g/day/mouse). All animals received water *ad libitum.* During the experiment, mice were weighted once a week.

The animals were housed in separated cages under controlled conditions of optimum temperature (21 °C) and light (12-hr light/12-hr dark cycle) until the age of 10 months, when animals were weighted and sacrificed with thiopental at a dose of 90 mg/kg body mass and the ovaries collected.

### Morphological analysis of ovarian tissue

At the end point of the experiment, the ovaries were isolated for histological analysis. They were then fixed in 4 % buffered formalin, embedded in paraffin, and sectioned at a thickness of 3 μm with a Microtome HM 325. The sections were mounted on glass slides, counterstained with hematoxylin and eosin (H&E), and examined by light microscopy (BX41 microscope, Olympus).

## Immunohistochemistry

### Immunohistochemical reaction of the ERs and the AR

Expression of the ERs and AR were determined immunohistochemically in paraffin-embedded specimens fixed in 4 % buffered formalin. The reactions were performed on deparaffinized and rehydrated sections, which were cut and mounted on microscope slides. The slides were heated in buffer pH9 (DakoTarget Retrieval, Denmark) for 30 min, and endogenous peroxidase was inhibited by Dako REAL peroxidase-blocking solution. Next, anti-estrogen receptor and anti-androgen receptor antibodies were applied (both purchased in Thermo Scientific, USA) for 30 min. To visualize the immunohistochemical reaction, Dako REAL EnVision Detection System Peroxidase/DAB/Rabbit/Mouse was used, and the sections were counterstained with Meyer’s hematoxylin. Finally, the sections were examined with an Olympus IX81 inverted light microscope (Olympus, Germany) with color camera and CellSens image-processing software (Olympus, Germany).

From each female (AL n = 6 and CR n = 6), 2 representative sections from each ovary (24 ovaries) were used for examination of ER and AR expressions.

### Quantitative image computer analysis of immunohistochemistry

All preparations were subjected to scanning procedure employing a ScanScope AT2 scanner (Leica Biotechnologies), at × 200 magnification (resolution 0.25 μm). The obtained digital images were analyzed on a computer screen using an ImageScope viewer (version 11.2.0.780, Aperio Technologies, Inc). The percentage of the AR-positive and ER-positive cells was calculated quantitatively for all studied types of ovarian cells using grades: −, +, ++ and +++. In order to obtain reliable and repetitive values, the total number of AR-positive and ER-positive cells was counted in 10 random fields in twelve slides for each group (control and experimental animal ovaries).

### Statistical analysis

Statistical analysis was conducted using the program Statistica 8.0 for Windows (StatSoft, Poland). The arithmetic mean and standard deviation were calculated for quantitative variables in each group. In order to assess normality distribution Shapiro-Wilk test was used. U Mann–Whitney test was performed to assess the significance of differences between the values obtained for each of the groups.

## Results

### Weight of animals and histology of ovaries

During the 9 months of experiments, animals from both groups gained weight. The final body mass of females in the CR group did not differ from the body mass of females in the group fed *ad libitum* (Table 1).

**Table 1:** The body mass of female mice of the CR group and the group fed *ad libitum* (AL)

Experimental group	Beginning [g] ± SD	End [g] ± SD	Mass gain [%]
CR female mice	22.14 ± 1.97	29.133 ± 2.46	31.58
AL Female mice	21.24 ± 1.15	30.133 ± 3.35	41.87

### Immunoexpression of the estrogen receptors (ERs) in ovary

**Fig. 1:**
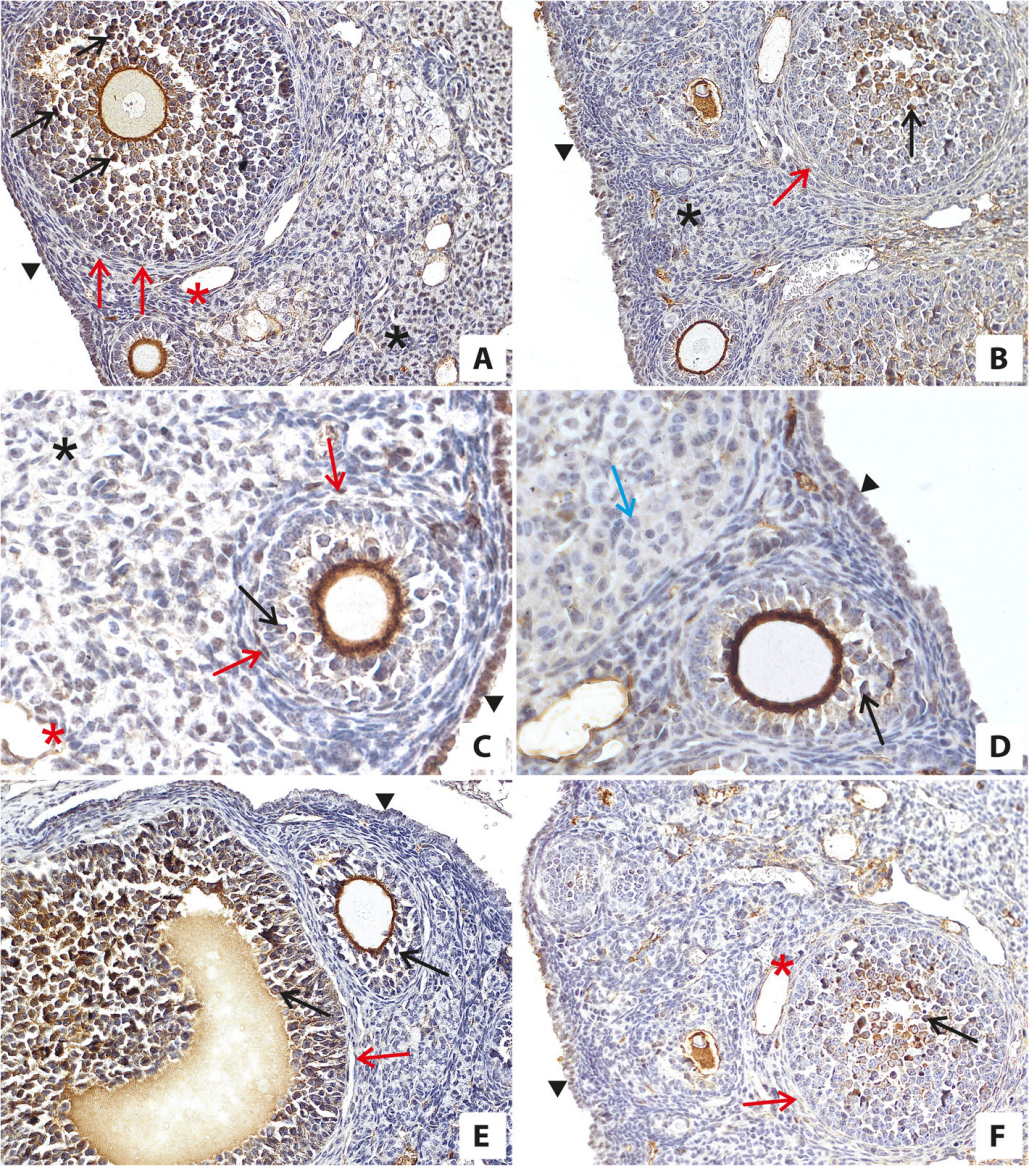
Immunolocalization and immunoexpression of ERs in the ovaries of mice on CR (**a**, **c**, **e**) and in the ovaries of mice fed *ad libitum* (**b**, **d**, **f**). The high expression of ERs was observed in ovaries of mice on CR in granulosa cells (black arrow), theca cells (red arrow), ovarian surface epithelium cells (black arrowhead), interstitial cells (black asterisk), and endothelial cells (red asterisk) (**a**, **c**, **e**) compared with expression of ERs in the ovaries of control mice fed *ad libitum* in granulosa cells (black arrow), ovarian surface epithelium cells (black arrowhead), interstitial cells (black asterisk), and endothelial cells (red asterisk) (**b**, **d**, **f**). There was no immunoreaction in theca cells (**b**, **f**; red arrow) and in luteal cells (**d**; blue arrow) in the ovaries of mice fed *ad libitum* (**b**, **d**, **f**). Objective magnification: **a**, **b**, **e**, **f**, x20; **c**, **d**, x40

In the ovaries of mice in the experimental group under CR, we found immunoexpression of ERs in granulosa cells, ovarian surface epithelium cells, endothelial cells, and interstitial cells and, in addition, very weak immunoexpression of these receptors also in theca cells and weak immunoexpression in luteal cells (Table 2, Fig. 1).

**Table 2:** Immunoexpression of estrogen (ER) and androgen (AR) receptors in the ovaries of mice on CR and fed *ad libitum*

Receptors	Ovarian Surface Epithelium	Granulosa cells	Theca cells	Luteal cells	Endothelial cells	Interstitial cells
Estrogen receptors						
Experimental group	+++	+++	+/−	+	++	++
Control group	+	++	-	-	+	+
Androgen receptor						
Experimental group	++	+++	+/−	-	++	+
Control group	++	+++	-	+	+	+

Legend: very strong reaction(+++), strong reaction(++), weak reaction(+), very weak (+/−), no reaction(−)

In the ovaries of animals in the control group, ERs were expressed only in granulosa cells, in ovarian surface epithelium cells, endothelial cells and interstitial cells and was weaker than in mice under CR (Table 2, Fig. 1).

### Immunoexpression of the androgen receptor (AR) in ovary

**Fig. 2:**
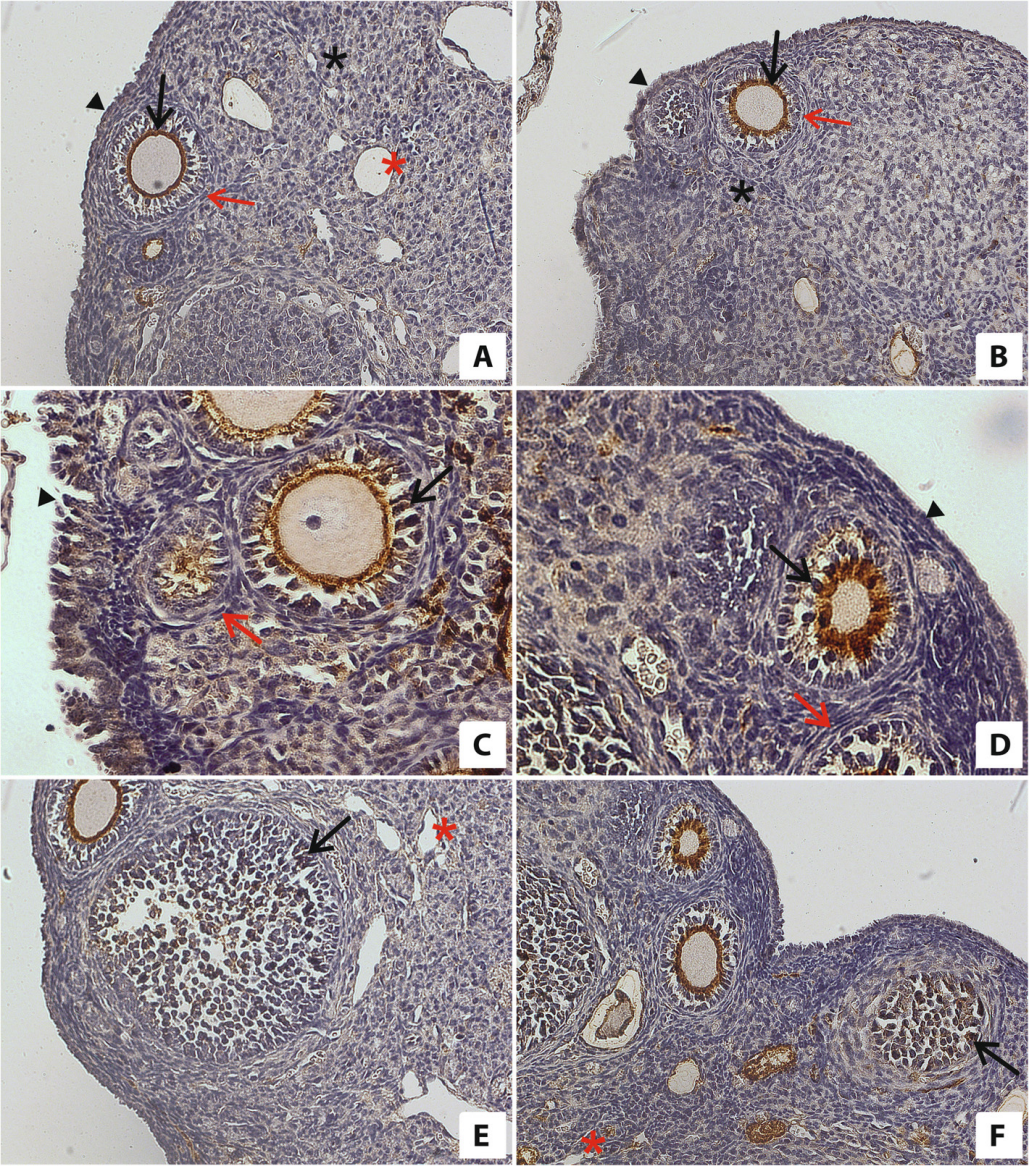
Immunolocalization and immunoexpression of the AR in the ovaries of mice on CR (**a, c, e**) and in the ovaries of mice fed *ad libitum* (**b**, **d**, **f**). One can see a similar expression of the AR in granulosa cells (black arrow) and ovarian surface epithelium cells (black arrowhead) in ovaries of mice on CR (**a**, **c**, **e**) and in ovaries of mice fed *ad libitum* (**b**, **d**, **f**). Expression of the AR in interstitial cells is shown by black asterisks in ovaries of mice fed *ad libitum* (**b**) and in the ovaries of mice on CR (**a**). Red asterisks indicate expression of AR in endothelial cells in the ovaries of mice fed *ad libitum* (**f**) and in the ovaries of mice on CR (**a**, **e**). There was no immunoreactions to the AR in theca cells (red arrow) in the ovaries of mice fed *ad libitum* (**b**, **d**). There was very weak expression of the AR in theca cells (red arrow) in the ovaries of mice on CR (**a**, **c**). Objective magnification: **a**, **b**, **e**, **f**, x20; **c**, **d**, x40

In the ovaries of mice in the experimental group under CR, we found expression of the AR in granulosa cells, ovarian surface epithelium cells, endothelial cells, and interstitial cells, and also in theca but not in luteal cells (Table 2, Fig. 2). By contrast, in the ovaries of control mice, we found expression of the AR in granulosa cells, luteal cells, ovarian surface epithelium cells, endothelial cells, and interstitial cells (Table 2, Fig. 2). Of note, we didn’t find expression of these receptors in theca cells in ovaries of the control group (Table 2, Fig. 2). Furthermore, in ovaries of experimental and control groups we observed a similar high level of expression of the AR in granulosa cells and in surface epithelium cells. In the ovaries of the experimental group on CR, we noted higher expression of the AR in endothelial cells than in ovaries of the control group. In the experimental group, we observed very weak expression of the AR in theca, weak expression in interstitial cells and a lack of immunoreactivity in luteal cells (Table 2, Fig. 2).

In ovaries of the control group, the immunoreactivity of the AR in interstitial cells was similar to the expression of AR in interstitial cells in ovaries of the experimental group. Furthermore, expression of the AR was lower in control mouse ovarian endothelial cells than in experimental mice. We also noted a weak immunoreaction of the AR in luteal cells in ovaries of control group females (Table 2, Fig. 2).

The immunohistochemical evaluation in all sections in all types of ovarian cells was confirmed by quantitative image analysis and expressed as percentage of positive cells. Results of quantitative analysis are presented in Table 3. Results indicated with asterix (*) are statistically significant (p < 0,05).

**Table 3:** The percentage of AR and ER positive cells in ovaries in CR and fed *ad libitum* mice. Data expressed as mean ± SD

Group		Control		Experimental	
		AR	ER	AR	ER
Ovarian surface epithelium	Lack of reaction (−)	9,2 ± 13,0	22,5 ± 4,2	10,3 ± 12,2	6,5* ± 8,5
	Weak reaction (+)	28,3 ± 11,7	52,9 ± 6,8	15,7* ± 13,4	15,1* ± 6,3
	Strong reaction (++)	62,0 ± 15,6	22,1 ± 7,1	56,3 ± 15,3	6,9* ± 3,5
	Very strong reaction (+++)	0,6 ± 1,8	2,5 ± 2,2	17,7* ± 6,6	71,5* ± 12,6
Granulosa cells	Lack of reaction (−)	10,0 ± 11,9	0,2 ± 0,7	13,6 ± 7,5	0,1 ± 0,4
	Weak reaction (+)	6,4 ± 3,1	9,5 ± 9,0	14,2* ± 5,9	2,3* ± 3,0
	Strong reaction (++)	26,5 ± 8,9	72,7 ± 10,4	25,9 ± 7,7	18,7* ± 6,0
	Very strong reaction (+++)	57,1 ± 10,9	17,6 ± 9,5	46,3* ± 6,2	79,0* ± 6,5
Theca cells	Lack of reaction (−)	89,4 ± 6,4	79,1 ± 6,8	34,8* ± 6,1	28,7* ± 10,7
	Weak reaction (+)	9,0 ± 4,9	11,3 ± 4,2	49,7* ± 6,5	50,2* ± 9,9
	Strong reaction (++)	0,5 ± 1,4	7,9 ± 4,6	11,1* ± 4,3	13,3 ± 6,0
	Very strong reaction (+++)	1,0 ± 3,2	1,6 ± 2,6	4,2* ± 4,9	7,7* ± 4,5
Luteal cells	Lack of reaction (−)	24,9 ± 7,8	76,8 ± 15,2	93,1* ± 1,9	33,8* ± 7,8
	Weak reaction (+)	73,7 ± 6,9	14,3 ± 7,3	6,9* ± 2,0	50,6* ± 5,5
	Strong reaction (++)	1,3 ± 0,8	8,3 ± 8,1	0,1* ± 0,2	14,1 ± 5,0
	Very strong reaction (+++)	0,1 ± 0,2	0,6 ± 0,8	0,1 ± 0,2	1,5 ± 1,6
Endothelial cells	Lack of reaction (−)	20,6 ± 9,6	20,8 ± 9,7	0,1* ± 0,2	17,7 ± 7,8
	Weak reaction (+)	70,4 ± 10,6	50,9 ± 7,7	21,0* ± 12,6	15,5* ± 8,4
	Strong reaction (++)	8,1 ± 4,8	25,9 ± 11,7	52,6* ± 5,2	47,1* ± 7,7
	Very strong reaction (+++)	0,8 ± 1,8	2,4 ± 3,2	26,3* ± 9,2	19,7* ± 12,9
Interstitial cells	Lack of reaction (−)	5,5 ± 8,3	24,0 ± 5,0	19,1* ± 11,3	6,3* ± 4,4
	Weak reaction (+)	72,4 ± 7,6	47,6 ± 5,8	79,4 ± 10,7	21,7* ± 10,3
	Strong reaction (++)	20,2 ± 11,6	22,8 ± 5,9	1,4* ± 1,5	56,8* ± 6,3
	Very strong reaction (+++)	1,8 ± 2,2	5,7 ± 3,9	0,1 ± 0,3	15,3* ± 9,7

*p < 0,05 vs control

## Discussion

It is known that caloric restriction prolongs life span in many species [21, 24, 26]. In our recent work, we reported that ovaries of mice fed *ad libitum* had smaller numbers of primordial, primary, and preantral follicles than ovaries of mice on CR [25]. Here, we wanted to check whether CR influences the expression of ERs or the AR in murine ovaries. This is an important question, because estrogens and androgens have a crucial role in the normal function of the reproductive system [1, 4–6, 12, 14, 15]. The salient observation of our study is an increase in expression of ERs in the ovaries of animals on CR. By contrast, in female mice of reproductive age, expression of the AR in ovarian cells did not change, regardless of whether mice were under CR or fed *ad libitum.* Specifically, we found similar expression of the AR in both groups of animals, particularly in granulosa cells and in ovarian surface epithelium cells. We also observed individual theca, interstitial, and endothelial AR-positive cells.

An increase in the level of androgens in females is associated with obesity, and androgens reduce hypothalamic sensitivity to steroid negative feedback [31]. In our experiment, we did not observe differences in weight between mice in experimental and control groups. Hułas-Stasiak et al. [19] found that expression of the AR decreases during follicular development. In our previous work we observed higher numbers of primordial, primary, and preantral follicles in ovaries of mice on CR than in the control group [25]. Since the development of follicles in these early stages depends on androgen levels [8, 12, 15, 16], one can speculate that follicular development is normal in the androgen-dependent stages of folliculogenesis in mice under CR and diminished in the estrogen-dependent stages due to decreased levels of estrogens. Because the early stages of follicular development were not influenced by CR, we assume that androgen levels during the CR experiment were not changed and that this was the reason for the lack of change in expression of the AR.

Moreover, a decrease in testosterone level due to dietary restriction together with premenopausal weight loss has been observed in women [32]. The lack of change in weight in our mice is probably related to a lack of change in expression of the AR, most likely because of a lack of change in androgen level. Other groups [33] found that expression of the AR did not change in ovaries of postmenopausal women, in spite of a permanent decrease in androgen plasma level. Interestingly, in other reproductive organs, it was shown that changes in expression of the AR do not depend on androgen levels in plasma [34].

In contrast to the lack of AR expression changes, we observed differences in expression of ERs in murine ovaries between experimental and control groups. Specially, we noted higher expression of ERs in granulosa cells and surface epithelium cells in ovaries of mice on CR compared with ovaries of mice fed *ad libitum*. In parallel, we also observed a slightly increased expression of ERs in theca, luteal, endothelial, and interstitial cells.

Nilsson et al. [35] reported that expression of ERα is reduced in adipocytes from obese female mice compared with normal-weight mice. Moreover, Foryst-Ludwig et al. [36] observed that ERβ knockout mice fed a high-fat diet had enhanced PPARγ signaling, increased fat mass, and improved insulin sensitivity. This study provided evidence for a role of estrogens in overweight conditions and obesity [36]. In our experiment, the expression of ERs in the ovaries of mice on CR increased, and we observed no reduction in body mass. This result is in accordance with results obtained in previous studies of humans and rodents concerning the influence of estrogens level on weight and the amount of adipose tissue [35, 36]. It has been demonstrated that, while estrogen treatment reduces adipose tissue mass in postmenopausal women, the decrease in estrogen levels after ovariectomy leads to an increase in adipose tissue mass [37]. To explain this finding, Mauvais-Jarvis [23] reported that ERs control energy intake and expenditure.

The ovarian expression of ERs is regulated by estrogen levels in blood plasma, and in ER knockout mice, estrogen and LH plasma levels are elevated [38]. This suggests the presence of an equilibrium between estrogen and LH levels and expression of ERs at the protein level [38]. In our study, we noted an increase in ER expression in the ovaries of CR female mice compared with littermates fed *ad libitum*. This result could potentially be explained by a decrease in estrogen level in blood plasma in animals on CR. A decrease in estrogen and LH levels in rodents and heifers on a CR diet was observed by Li and Hussein [39, 40]. Similar results were obtained by Williams et al. [41] in a study of premenopausal women on CR and exposed to exercise.

In our experiment, we did not observe a decrease in the body mass of female mice after 9 months of CR. It is known that after menopause or ovariectomy in humans the decrease in estrogens is paralleled by an increase in body weight [23]. We assume that the lack of a decrease in body weight in our mice under CR is due to a decrease in estrogen levels, which leads to a decrease in energy expenditure in female mice. This conclusion, however, requires further study, including direct measurement of estrogen levels in blood plasma. We also noted increased liver weight and steatosis in female mice on CR compared with control mice, which can be linked to a decrease in energy expenditure and suggests energy savings (unpublished data). In support of this possibility, Rogers et al. [42] observed hepatic steatosis and a decrease in energy expenditure in ovacteromized mice in which the estrogen level decreased. Decreased production of estrogens may be linked to decreased activity of aromatase P450, which is regulated by IGF-1 level [43]. In fact, the decreased level of IGF-1 in plasma during CR in female rats was confirmed by Li et al. [44]. It is also possible that fat depots in CR females are bigger than in control animals that were fed *ad libidum*. Even if we did not notice increase in body weight in animals under CR the potential re-distribution of fat tissue requires further studies.

In conclusion, prolonged CR (9 months) in female mice leads to increased expression of ERs, while at the same time it does not affect expression of the AR in ovarian cells. Caloric restriction results in lower level of estrogen in circulating blood which leads to higher sensitivity of cells to this hormone. We envision that return to normal diet after period of CR increases number and enhances maturation and function of follicles due to improved responsiveness of ovarian tissue to estrogens action and may be helpful in reproductive success.

## Abbreviations

CR: caloric restriction
AR: androgen receptor
ERs: estrogen receptors
FSH: follicle stimulating hormone
LH: luteinizing hormone
IGF-1: insulin-like growth factor 1
WT: wild type
